# Oral examination as a tool for mastery learning in undergraduate microbiology

**DOI:** 10.1093/femsle/fnag010

**Published:** 2026-01-24

**Authors:** Andrew R St James, Camille Widener

**Affiliations:** Department of Biology, Wake Forest University, Winston-Salem, NC 27101, United States; Department of Biology, Wake Forest University, Winston-Salem, NC 27101, United States

**Keywords:** oral examination, *viva voce*, mastery learning, microbiology education, assessment techniques, undergraduate education

## Abstract

Undergraduate courses in general microbiology often have high content and cognitive loads, making it challenging for students to achieve content mastery of the whole curriculum. Thus, the development of manageable mastery learning interventions that work within these contexts should benefit microbiology education broadly. In this work, we describe an oral examination intervention implemented within a modified Bloom’s Learning for Mastery framework that significantly improves retention of course material across low-performing students in an undergraduate general microbiology course. The intervention consumed a relatively small amount of instructor time to administer (less than 15 min per student) and resulted in no distinguishable differences in knowledge retention on the final exam between students who initially mastered the content and did not participate in the intervention and those who participated in the intervention after failing to initially master the content. Students reported overwhelmingly positive experiences with the intervention, including increased perceptions of their own content retention, increased feelings of self-pride after participating in the intervention, and a general feeling that the intervention was fair. We conclude that oral examinations are an effective mastery learning tool in the microbiology classroom and can be easily implemented by the instructor alone in small- to medium-sized courses.

## Teaser

An oral examination intervention improves content retention in low-performing microbiology students.

## Introduction

Students in microbiology courses often struggle to retain knowledge due to the large amount of content that is covered (Struwig et al. 2016, Boury et al. 2024), and large content load may also pose a barrier to implementation of evidence-based teaching approaches that could help students retain content (Horak et al. 2015, Petersen et al. 2020). Despite these challenges, many scholars and learned societies in microbiology agree on the importance of microbiology literacy for making informed personal and collective decisions about microbiology-related issues (e.g. pandemic preparedness, environmental quality, energy production, food safety, etc.) (Merkel 2012, Fahnert 2017, Carvalho and Lima 2022, Timmis et al. 2024), especially in an age of rising global disinformation (West and Bergstrom 2021, Oliveira et al. 2022, Dobson 2024).

Mastery learning is an instructional philosophy predicated on the belief that all students are capable of “mastering” any given material if provided sufficient time and resources (Block and Burns 1976, Slavin 1987). Since its inception in the 1960s by Benjamin Bloom, numerous instructional strategies have been developed for implementation within mastery learning models (Slavin 1987), the most widespread of which is Bloom’s Learning for Mastery (LFM) model (Bloom 1968, Block and Anderson 1975). In this model, the instructor administers a formative assessment to all students covering a unit or module’s content and establishes a criterion for mastery (typically 80%–90%). Students who do not achieve mastery receive corrective instruction and a subsequent parallel formative assessment. Typically, students continue this revision and re-assessment process until they have successfully mastered the content.

Research has shown that students instructed within mastery learning models often perform better academically than students in non-mastery learning models (Kulik et al. 1990, Winget and Persky 2022). Thus, mastery-based strategies should be viewed as fruitful avenues for exploration of classroom strategies that achieve high scientific literacy rates in high content area courses like microbiology. One barrier to implementing these strategies however is instructor time (Arlin 1984, Álvarez et al. 2025), especially if instructors are to provide individualized feedback to students (Mandernach 2018), which is critical to achieving mastery learning goals (Eppich et al. 2015). Here, we propose that oral examinations may be an effective strategy that minimizes instructor time while maximizing student evaluation and feedback potential.

Oral examination, or “*viva voce*,” is a robust assessment strategy for the undergraduate classroom. While oral examinations have been historically underutilized in undergraduate education, their popularity is increasing in response to post-COVID 19 changes to the educational landscape (Brogan et al. 2024, Curbo et al. 2025), including threats of rising academic dishonesty in the face of Generative Artificial Intelligence (GenAI) tools like ChatGPT (Akkaraju 2023, Mariano et al. 2024). From the instructor perspective, a major benefit of the oral examination is the ability to more thoroughly assess understanding by probing student explanations (Roecker 2007, Theobold 2021) and then to provide individualized explanatory feedback to correct student misconceptions and to fill continuing knowledge gaps (St. James and Campbell 2020). As a mastery learning tool, oral examination is relatively unexplored, though broader research on testing as a learning tool has suggested that testing may be superior to re-studying for promoting long-term retention (Rawson et al. 2013, Vaughn et al. 2016, Yang et al. 2019).

In this study, we designed an oral examination intervention and implemented it within a modified Bloom’s LFM framework. Briefly, students were graded for mastery on written assessments, and students who failed to attain mastery were given the opportunity to go through a subsequent process of oral examination to attain mastery. We tracked the performance of students who did and did not participate in the intervention to evaluate their content retention and collected data on student perceptions of the intervention. We hypothesized that students going through the intervention would significantly improve their content retention relative to those who did not.

## Materials and methods

### IRB approval

This research was approved as a minimal risk project by the Institutional Review Board (IRB) of Wake Forest University under IRB Number: IRB00025871.

### Course structure and participants

This study was conducted at a R2 Carnegie classification university in the USA during the Spring semester of the 2024–2025 Academic Calendar Year. The course of study was an advanced undergraduate-level microbiology course taken primarily by Biology majors and minors. The course carries as pre-requisites two semesters of introductory biology with laboratory and one semester of organic chemistry. Twenty students were enrolled in the course and all students agreed to participate in the study. Fifteen of the students registered for the course were biology majors and five were biology minors.

The course was divided into five modules, each consisting of three or four lectures and a paper discussion of a recent primary literature article. Classes met twice weekly for 75 min each throughout a 15-week semester (35 total semester hours of lecture). Students were provided with guided notes to accompany each lecture, which were subdivided into “Content Blocks” denoted by bold and underlined headings. The assessment was based on a modified Bloom’s LFM model. Students began by taking a traditional written exam covering the content from the module lectures and paper discussion. Exams were graded for mastery with a cutoff of 80% overall based on an average of points across each individual question on the exam. For simplicity while grading individual questions, points were awarded for individual exam questions in quarter-point increments. If a student’s overall exam score was considered to have reached a level of mastery, they received a grade of “Satisfactory” on the exam and continued to the next module without a grade penalty (i.e. if their grade before the exam was an A, it remained an A). Otherwise, the student received a grade of “Unsatisfactory” on the exam and was allowed to enter the intervention phase of assessment to prevent incurring a grade penalty. If a student chose not to participate in the intervention or was still performing unsatisfactorily after the intervention, they incurred a grade penalty of a single grade point (i.e. if their grade was an A before the exam, it was an A^−^ after the exam). All assignments in the course were graded with the same mastery-based scheme described above and a student’s overall course grade was determined by the total number of “Unsatisfactory” grades remaining in the gradebook at the end of term. Thus, all course assignments held equal weight.

The intervention phase consisted of up to two oral examinations re-assessing any material covered within a Content Block relevant to a question for which the student did not receive full credit on the exam (e.g. if a student did not receive full credit on a question relating to the role of RNA polymerase in transcription, they were required to retest on *all* material related to transcription during the oral examination). Students could schedule their oral exams at any time during the semester, with the deadline for retakes being set as the last day of classes, though the instructor encouraged students to at least begin the retake process within one week of receiving an unsatisfactory grade. If a student attended office hours after the exam to review material, they were allowed two re-assessment opportunities. Otherwise, students were only allowed a single re-assessment opportunity. Regardless of whether a student had exhausted their re-assessment opportunities or not, they were provided detailed, individualized feedback on their content mastery after each oral examination. While a classical application of Bloom’s LFM model would allow students an unlimited number of opportunities to continue re-testing, practical considerations around instructor time necessitated the limitations described above.

Oral examinations took the form of semi-structured question-and-answer sessions between the instructor and the student. Efforts were made to administer individualized exams while maintaining exam validity and reliability based on established best practices (Memon et al. 2010). To ensure validity, all exam questions focused on learning objectives that were provided to students in advance. Questions were not regurgitations of written exam questions, which were narrow and specific, rather, they were generally broad questions that would allow students the opportunity to describe whole pathways, processes, or concepts. To ensure reliability, students were initially asked the same questions for the same content. For example, students re-testing on the topic of bacterial transcription might be asked: “Describe the molecular events occurring during transcription initiation in bacteria.” To minimize subjectivity in grading, all students were required to demonstrate mastery of each topic, and the instructor pre-determined a list of required points a student would need to make to demonstrate mastery on each topic. For the example given above, students might be required to accurately describe the binding of sigma factor to the promoter sequence, the formation of the transcription bubble, and the release of sigma factor after initial nucleotides are added to the transcript. If a student did not address each point during the exam, the instructor then followed up on student answers by asking targeted follow-up questions based on student responses to probe potential knowledge gaps. For example, if a student mentioned that the RNA polymerase holoenzyme bound to the DNA but did not mention the sigma factor or the term promoter, they might be asked: “Can you describe the structure of the holoenzyme and highlight which component binds the DNA?” and “What is the term that we use for the region of DNA that is bound by RNA polymerase?” At the end of the exam, students were provided with explanatory feedback on their performance and outcomes were determined using the same grading scale as with the written exam. Explanatory feedback was provided using Hattie and Timperley’s Feed Up, Feed Back, and Feed Forward Model (Hattie and Timperley 2007). Additional examples and training materials from a presentation on developing and administering oral exams presented by the lead author at the American Society for Microbiology Conference for Undergraduate Educators (ASMCUE) meeting in Phoenix, AZ, USA in November 2023 can be accessed in Supplemental Text 1 (presentation slides) and Supplemental Text 2 (sample exam scripts).

### Study design and data analysis

To assess the effectiveness of the oral examination intervention, students were administered a final examination that included questions from previous exams. This section of the final exam contained eight questions, two from each module preceding the final exam. The final exam period consisted of both the Module 5 exam and the final exam described herein. Because the Module 5 exam was administered during the final exam period, only Modules 1–4 were able to be evaluated for oral exam effects. After the final exam period, students were still given the opportunity to engage in an oral retake of the Module 5 exam up until the day grades were due to the university, but retention data for this module was not able to be collected post-hoc. Students were informed the week before the exam that there would be a cumulative portion of the exam for which they could earn extra credit towards their final course grade. However, students were unaware this portion of the exam would consist of previous exam questions, and they did not have access to their previous exams while studying (previous exams were retained in the instructor’s office).

For students who initially received satisfactory grades on the exam, two questions from each initial exam were chosen at random using a random number generator (https://www.random.org). For students who initially received unsatisfactory grades on the exam, one question the student mastered was randomly chosen (if possible) and a second question the student did not initially master but had the opportunity to retest on was randomly chosen. This allowed us to compare whether the effect of the oral exam intervention extended to content the student did not retest on.

To evaluate retention of content mastery, responses to questions were divided into three groups: (i) questions students initially mastered (>75% of points for question) and did not have to revisit (“S” group), (ii) questions students initially did not master (<75% of points for question) but eventually mastered during an oral examination (“U to S” group), and (iii) questions students initially did not master and never successfully mastered (either because they chose not to retest on the material or they exhausted their retest opportunities without mastering the content) (“U” group). Statistical differences between initial and final exam performance on each question were determined using paired *t*-tests for within group comparisons and using two-sample equal variance *t*-tests for across group comparisons.

To evaluate student perceptions surrounding the oral exam intervention, a Qualtrics survey was administered (Supplemental Text 3). This survey consisted of Likert-scale and open-ended questions aimed at understanding student perceptions of exam stress, knowledge retention, and feelings of accomplishment.

## Results and discussion

### Effects of the intervention

**Figure 1:**
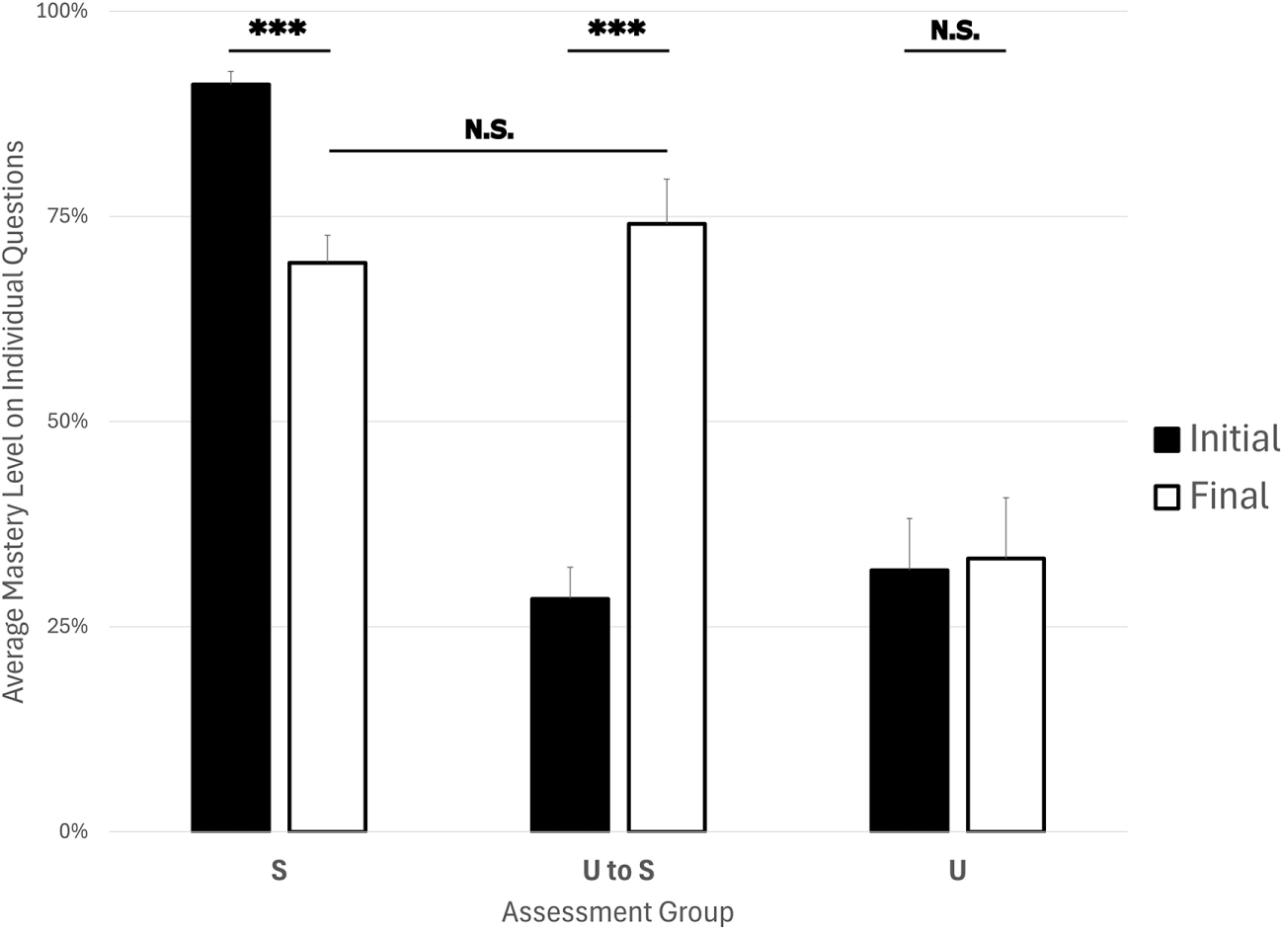
Students undergoing an oral examination intervention significantly improve their content mastery. Average mastery level on exam questions during initial assessment (black bars) and final exam assessment (white bars) for students who initially mastered the material (S Group), initially performed poorly but mastered the material after the intervention (U to S group), and never mastered the material (U Group). Error bars represent standard error of the mean. Significance levels: *P* < 0.05 (*), *P* < 0.01 (**), *P* < 0.001 (***), not significant (N.S.).

Of the twenty students enrolled in the course, 18 students (90%) took at least one oral exam. Averaged across four modules, scores on student answers to questions in the S group decreased (*P* < 0.001), whereas scores on student answers to questions in the U to S group significantly increased (*P* < 0.001). Scores on student answers to questions in the U group saw no change (*P* > 0.05) (Fig. 1). This overall trend was observed within individual modules as well (Supplemental Fig. 1). These data support our hypothesis that the oral examination intervention would lead to increased retention of course content.

The improved content retention in the U to S group questions is consistent with previous research demonstrating that repeated retrieval improves long-term retention (Karpicke and Roediger III 2007, Larsen et al. 2009). While few studies have evaluated oral examination as a retesting strategy, one study in a Calculus II course demonstrated that optional oral examinations before a summative assessment improved content retention in at-risk students (Nelson 2010).

Students were informed one week before the final exam that there would be a cumulative portion to the exam for extra credit, which all students were required to take. It is possible, however, that students answering questions in the U to S group prepared more for this portion of the exam than students answering questions in the S group. Several students answering questions in the S group performed highly on all course assessments throughout the semester and thus did not need to perform well on the extra credit portion of the exam to end up with a good final course grade. This likely led to an underperformance on questions in this group relative to the U to S group. This loss of content knowledge in the S group is unsurprising given that students may not have revisited the material before the final exam, and moderate content loss over time in the absence of repetition is a well-documented phenomenon (Custers 2010, Csaba et al. 2025). Nevertheless, despite the declines in retention for the S group questions, there was no statistically significant difference (*P* > 0.05) between the final scores on S group questions compared to the U to S group questions, suggesting that by the end of the semester, the performance of students who initially mastered the material relative to those who took advantage of the intervention was indistinguishable. The gains in the U to S group remain notable, suggesting that restudying the material after the oral examination intervention was sufficient to improve their retention. The fact that no students reviewed their prior exams before the final exam and were unaware that the re-tested questions would be regurgitations of the previous exam questions further supports our conclusion that content retention in the U to S group is legitimate.

Within the S group questions, data can be subdivided based on the overall exam performance of students. The first group (Group A) consists of students who performed well on the randomly selected question and received a Satisfactory overall exam score (i.e. they did not have to go through the re-testing protocol). The second group (Group B) consists of students who performed well on the randomly selected question but received an unsatisfactory overall exam score (i.e. they had to go through the re-testing protocol but did not have to re-test on the material related to the question that landed them in the S group). By comparing these two groups, we can evaluate whether retention of previously mastered material is influenced at all by revisiting different material within the same module. While Group B performed slightly higher than Group A overall (Supplemental Fig. 2), this difference was very small and not statistically significant (*P* > 0.05). This trend was also consistent within individual modules (Supplemental Fig. 3).

Bearing in mind that there is some loss of content knowledge in the S group, it may be worth considering whether to provide some form of re-testing to all students. Little research has been conducted on re-testing of students who have already mastered benchmarks, though a recent study performed within an introductory biology class for non-majors demonstrated that students with higher scientific reasoning ability performed better on a subsequent retention exam when they encountered non-cumulative testing throughout the semester, whereas low reasoners performed better when they encountered cumulative testing (Bailey et al. 2021), though it was unclear exactly why. While we are limited in our ability to draw direct comparisons between our groups and Bailey’s groups as we did not directly assess students’ scientific reasoning abilities, it is worth noting that exam questions were typically written to assess higher levels of Bloom’s taxonomy (e.g. analysis, synthesis, evaluation) (Bloom et al. 1956). Thus, landing is the S group for a given question may imply high scientific reasoning abilities in that question’s domain, though future work would need to be conducted to confirm this speculation. If true though, these findings may support continuing the re-testing intervention for the low performing students without the need for modifying or expanding the intervention to include initial high performers, saving instructor time and effort.

### Student perceptions of the intervention

**Figure 2:**
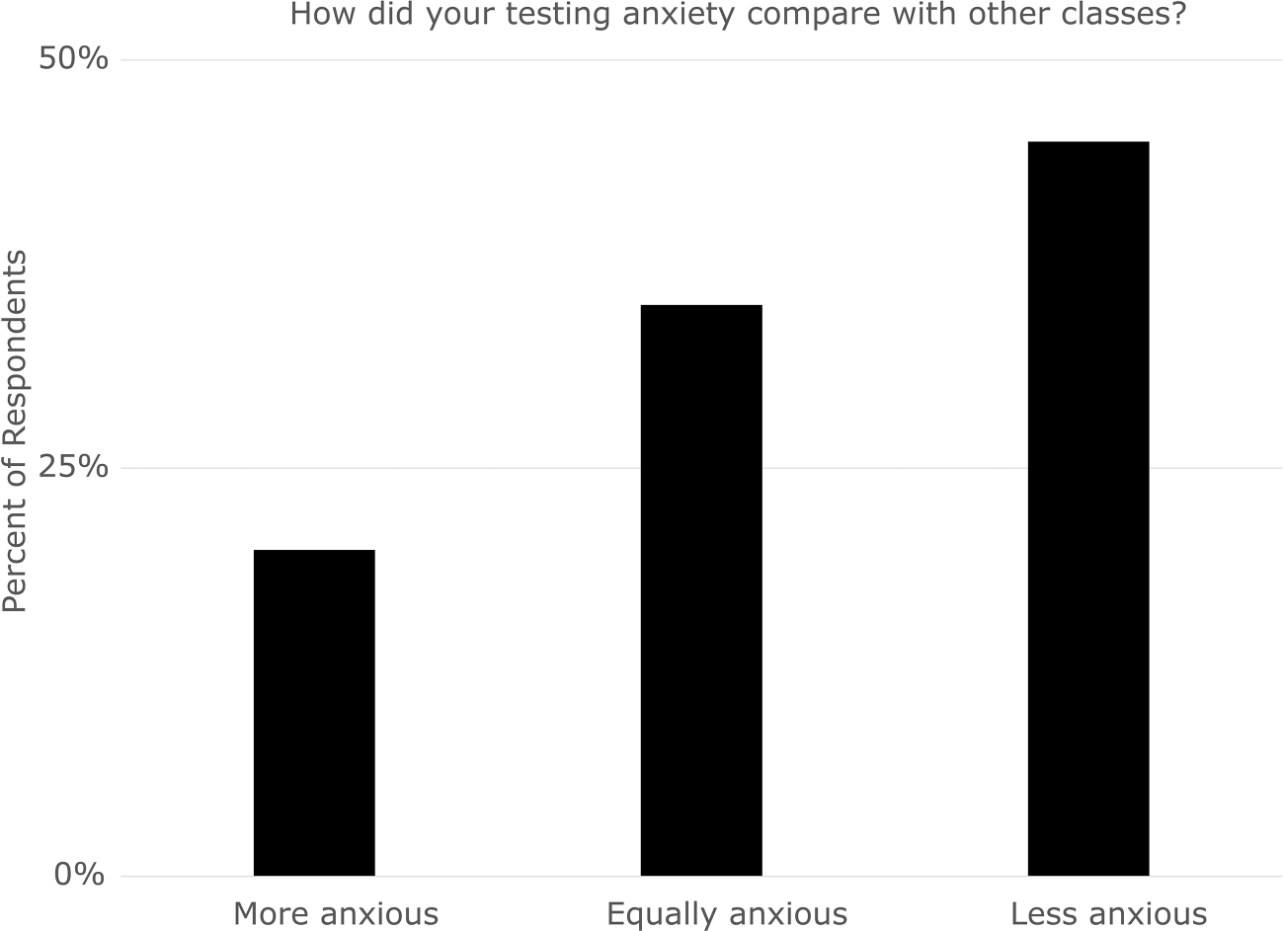
Most students report equal or lowered initial testing anxiety when they know the oral examination intervention is available. Percent of respondents to the question “How did your anxiety around written tests in Microbiology compare to your anxiety in other classes?”

We first asked students how their anxiety around the original written exams compared to their testing anxiety in other classes when they knew the oral retake intervention existed. While a minority of students were more anxious about testing in general, most students (80%) reported being either equally anxious or less anxious than in other classes (Fig. 2). We did not ask students to self-report their overall level of anxiety, only whether they felt a relative change in anxiety level knowing the intervention was there. For this reason, we cannot adequately evaluate whether students whose anxiety was unchanged actually exhibited testing anxiety in the first place or whether they were not anxious about testing in either case.

All the students who were more anxious attributed their increased anxiety (at least in part) to the level of detail and volume of content covered in the course relative to their other courses (Supplemental Table 1). Previous research supports that students are more likely to deem classes “hard” when they have high workload and high cognitive demand (Wyse and Soneral 2018), which may lead to increased anxiety. Of the students who were less anxious, two-thirds attributed their decreased anxiety to the retakes, while others attributed their decreased anxiety to other course attributes (e.g. guided notes, flexible exam scheduling) (Supplemental Table 2).

**Figure 3:**
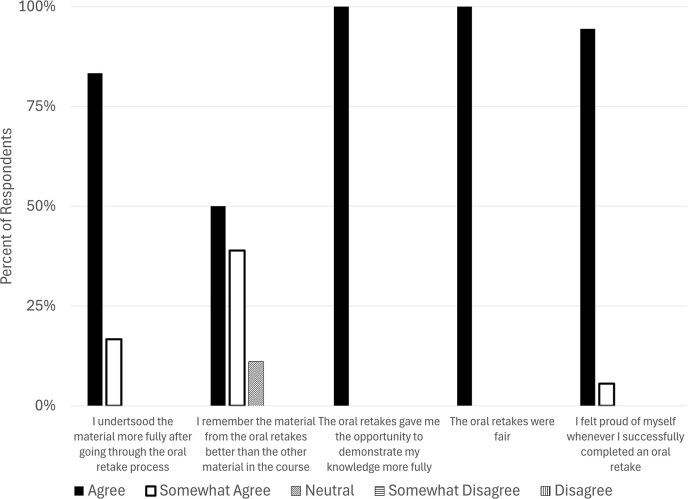
Students felt the oral examination intervention improved their content knowledge, was fair, and allowed them to feel pride in themselves. Likert-scale agreement to statements about the oral examination intervention.

Overall, 18/20 students in the course took at least one oral retake during the semester. We asked those 18 students further about their level of agreement with a series of statements about the oral exam (Fig. 3). All students agreed to at least some extent that they understood the material more fully after going through the retake process and most students (16/18) also agreed to at least some extent that they remembered material from the oral retakes better than the other material in the course. Encouragingly, 18/18 students fully agreed with the statements “The oral retakes were fair” and “The oral retakes gave me an opportunity to demonstrate my knowledge more fully,” and 18/18 students agreed to at least some extent with the statement “I felt proud of myself whenever I successfully completed an oral retake.”

When asked what they liked or disliked about the oral exam retake process, most students only reported positive items (Supplemental Table 3). In particular, several students reported that the exams helped them demonstrate what they knew better (e.g. “I think my knowledge finally was really expressed when I started doing them”) and others remarked on the quickness and directness of the retakes (e.g. “I thought they were quick and to the point[,] which I appreciated”). Four students reported that the oral exam was intimidating or stressful for them; however, all but one of these students qualified their responses to state that they still found value in the exam [e.g. “It was nerve racking (*sic*) to have to do an oral retake, as the stakes felt higher than a written exam. But from my one oral retake, I found that I slightly liked them better as I could early (*sic*) explain the concepts I missed.”]. This data is consistent with previous data indicating that some students may prefer oral assessments to written assessments (Huxham et al. 2012).

It is unsurprising that some students reported anxiety about the oral examination. Testing anxiety is widespread (Steinmayr et al. 2016) and both written and oral examinations have been shown to increase levels of salivary and plasma cortisol in students (Lacey et al. 2000, Schoofs et al. 2008, Maduka et al. 2015). Nevertheless, after considering the improved performance data from students after the oral examinations (Fig. 1), the fact that most students either enjoyed the exams [e.g. “Liked it all, thought it was fair and help (*sic*) me accountable”] or at the very least found utility in them, and reports of self-pride following successful exam performance, it suggests that the intervention does not present a barrier to learning for most students. In the small number of instances where students have documented accommodations to deal with stress or the instructor deems the exam too stressful for a given student, alternative retesting formats (e.g. a second written exam) might be considered.

### Limitations and considerations

One of the major limitations of this study is sample size (*N* = 20 students). However, given the size of the difference in performance between the groups (Fig. 1) and the extremely small *P*-values (all significant values had *P*-values below 0.001), we believe the results of this study are valid and could still be potentially applicable outside of the environment of a small class size, though further study may be warranted. While the first author used oral exams as a re-testing option for each of the seven semesters preceding this study, the semester described herein was the only semester for which IRB-approved data collection occurred. Anecdotally, we confirm that any time informal data collection has been conducted in previous semesters, the data have always been consistent with those presented herein. If these methods were to be implemented more broadly across different institutions with larger class sizes, analysis of data from those students could broaden our findings.

One potential barrier to implementation of oral exams is time—or rather, the perception that oral exams are more time intensive than other forms of assessment. By targeting oral retakes to only those topics that students did not initially master, we were able to constrain individual oral exams (including time for feedback) to no more than 15 min per student, rarely amounting to more than 2 h of instructor time per week. Others have also shown oral exams can be effectively administered within 15-min time frames (Morrissett 1958, Akkaraju 2023). Overall, we did not find the implementation of this intervention to be overly burdensome to the instructor’s time. A standard re-test rate in the course was around 40% of students. For a class of 20 students, this amounted to around 8–12 oral exams per module when taking into account both first and second retakes, resulting in an average of 3 h of instructor time every couple of weeks for administration of the exams. In larger classes, additional considerations would need to be made to administer oral exams; for example, graduate and undergraduate teaching assistants may be used to increase the throughput of oral exam implementation, if they are properly trained (St. James and Campbell 2020). In these courses, we suggest maintaining a student-to-instructor ratio of approximately 20:1 for feasible implementation of the exams.

A second barrier to implementation of oral exams is concern over maintaining fairness and equity across exams. This may be especially challenging if implementing the exams in a course with multiple instructors. To reduce biases across different instructors, all instructors could be asked to participate in implicit bias training before administering exams. These types of trainings, even short trainings, have been shown to heighten awareness of implicit biases in instructors over time (Sabin et al. 2022, Lindvall-Östling 2024). Additionally, exams may be monitored to reduce language barriers by providing students with clear indications of which specialized terms are required to be known (Memon et al. 2010). Re-wording of prompts during the oral examination can be provided to all students if they do not understand terms that are not required terms. Finally, while we acknowledge that implicit biases may remain, we propose that the structural aspects of our intervention may help mitigate the effects of these biases. Previous research has highlighted that structured activities designed to improve performance over time lead to reductions in achievement gaps between students (Freeman et al. 2011, Theobald et al. 2020). These practices are widely considered standards of equity-minded instruction (Artze-Vega et al. 2023). We therefore suggest that the combination of defined standards for mastery on each topic, structured and consistent feedback delivery, and policies enabling multiple retakes may maximize assessment equity through focusing instructor evaluation on the content rather than characteristics of the examinee.

## Conclusion

Overall, our data strongly suggests that our oral examination intervention was successful and that students significantly improved their content mastery because of retesting through the oral examination process. Importantly, scores from students who originally mastered the material were indistinguishable from those who encountered the intervention at the end of the semester, highlighting that the oral examination intervention explained herein was equitable. Student perceptions of the intervention were overwhelmingly positive and reports of stress from some students were mitigated by feelings of self-pride after completing the intervention and were typically accompanied by positive reflection on the overall utility of the exam for learning. We therefore suggest that oral examinations can be a useful tool in the arsenal of mastery learning strategies in the undergraduate microbiology classroom.

## Supplementary Material

fnag010_Supplemental_File
