# Supplementary material for: Oral examination as a tool for mastery learning in undergraduate microbiology
Source: FEMS Microbiol Lett. 2026 Jan 24;373:fnag010. doi: 10.1093/femsle/fnag010 (PMC13130396; doi:10.1093/femsle/fnag010)
Supplement: fnag010_Supplemental_File [file fnag010_supplemental_file.docx]

**Supplemental Files for “Oral Examination as a Tool for Mastery Learning in Undergraduate Microbiology**”

Andrew R. St. James^1*^, Camille Widener^1^

^1^Department of Biology, Wake Forest University, Winston-Salem, NC, USA

**Supplemental Text 1**: Slides from training presentation on designing and implementing oral exams presented at ASMCUE 2023 in Phoenix, AZ, USA.


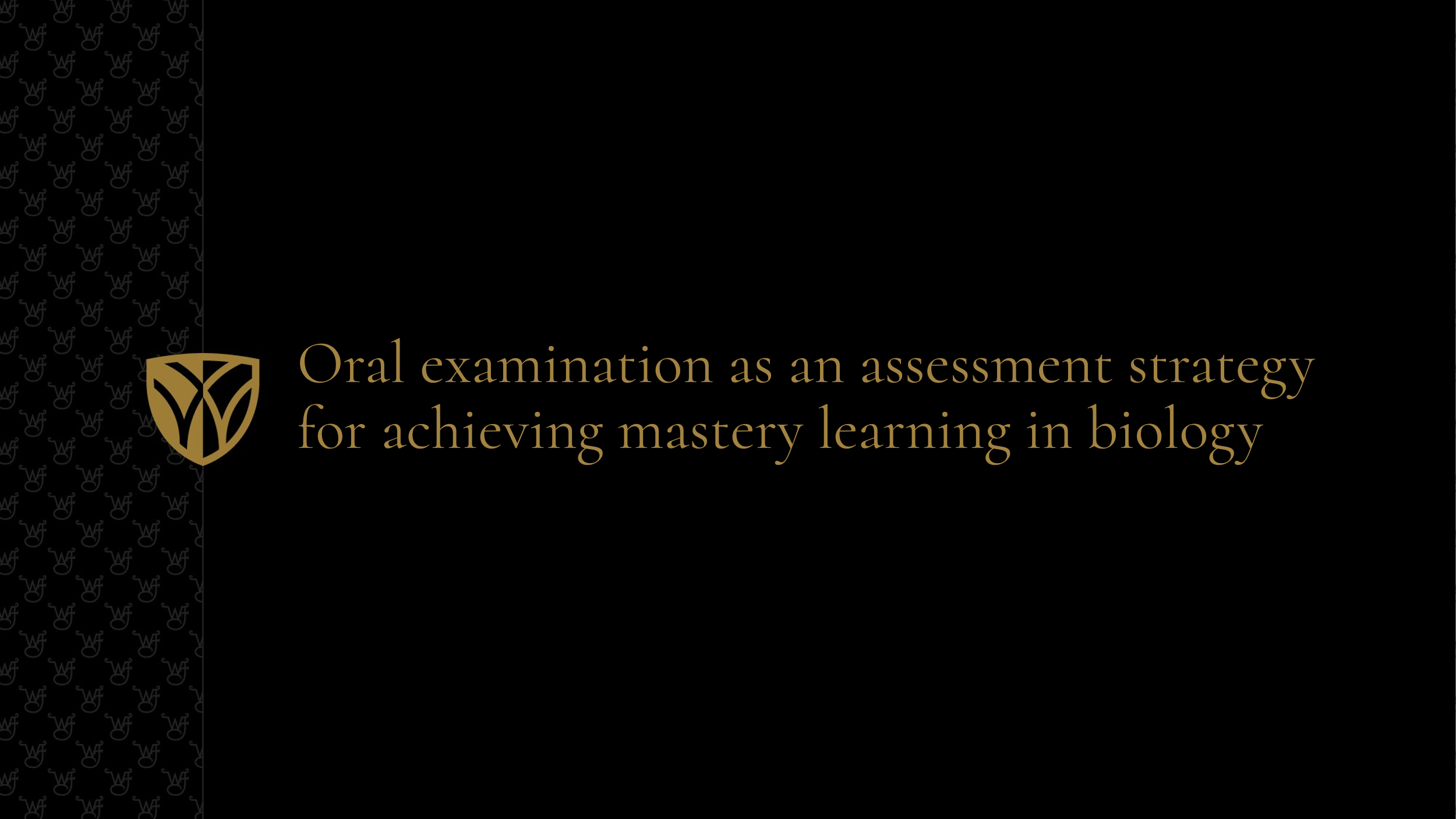


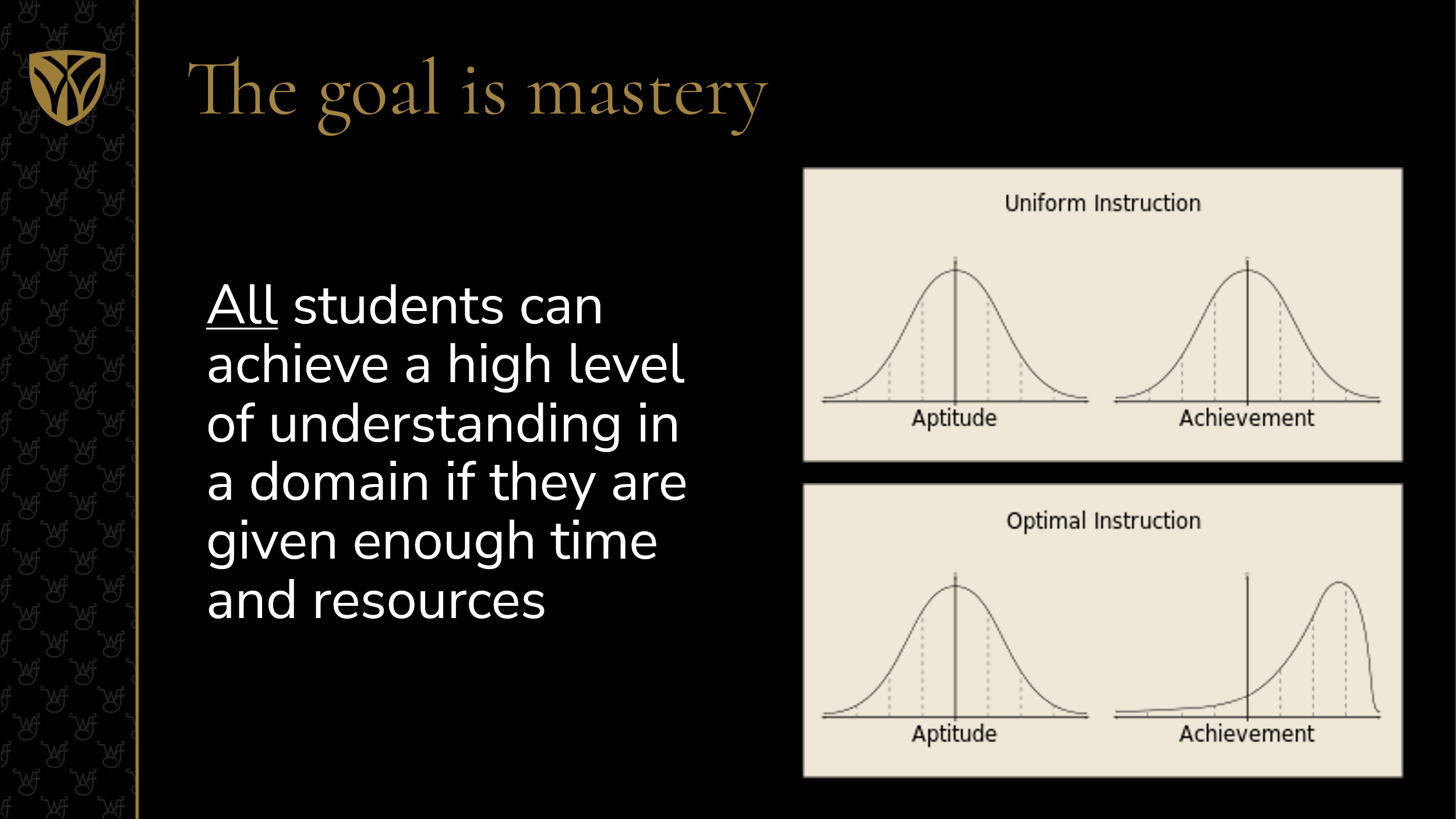


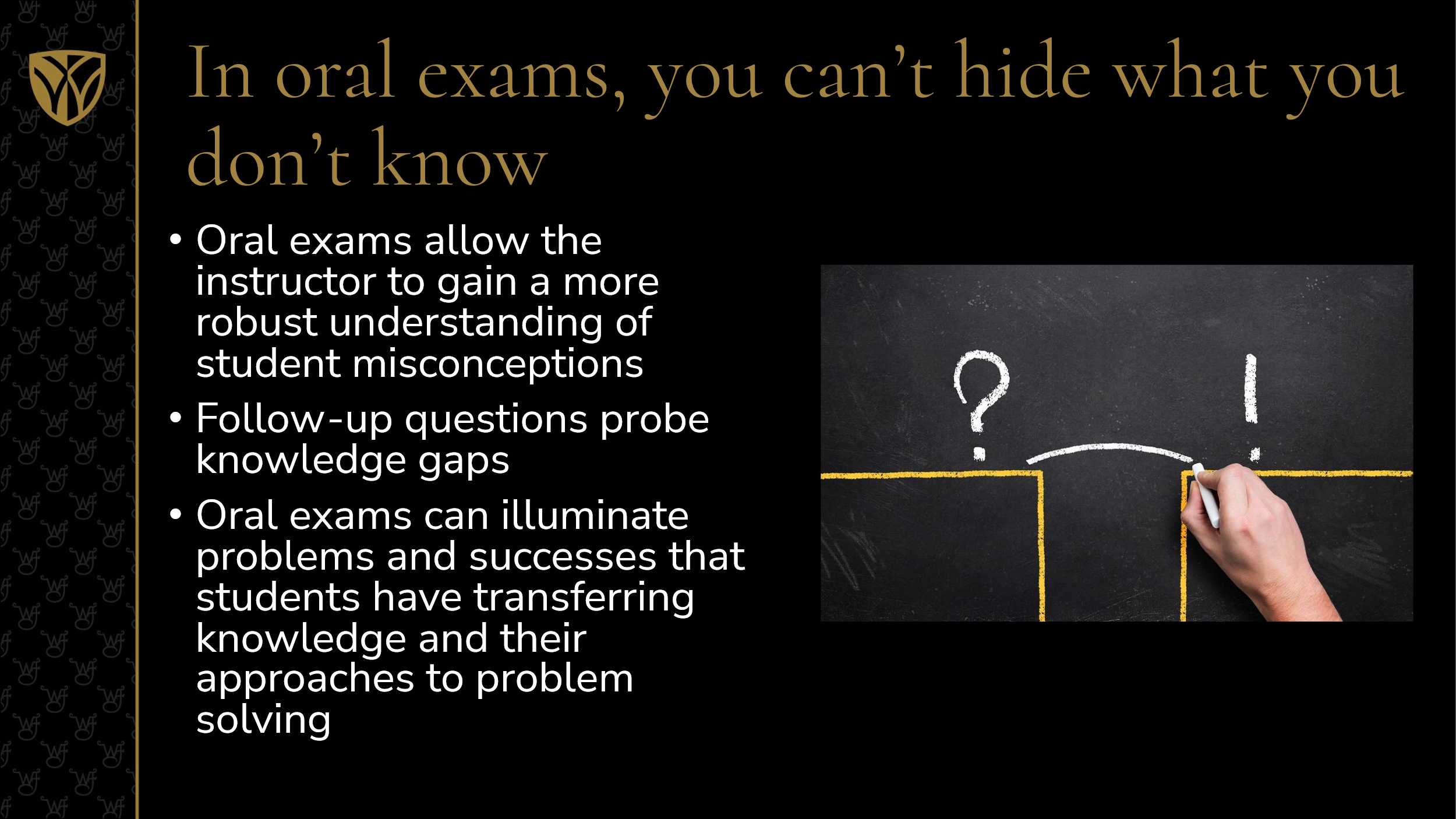


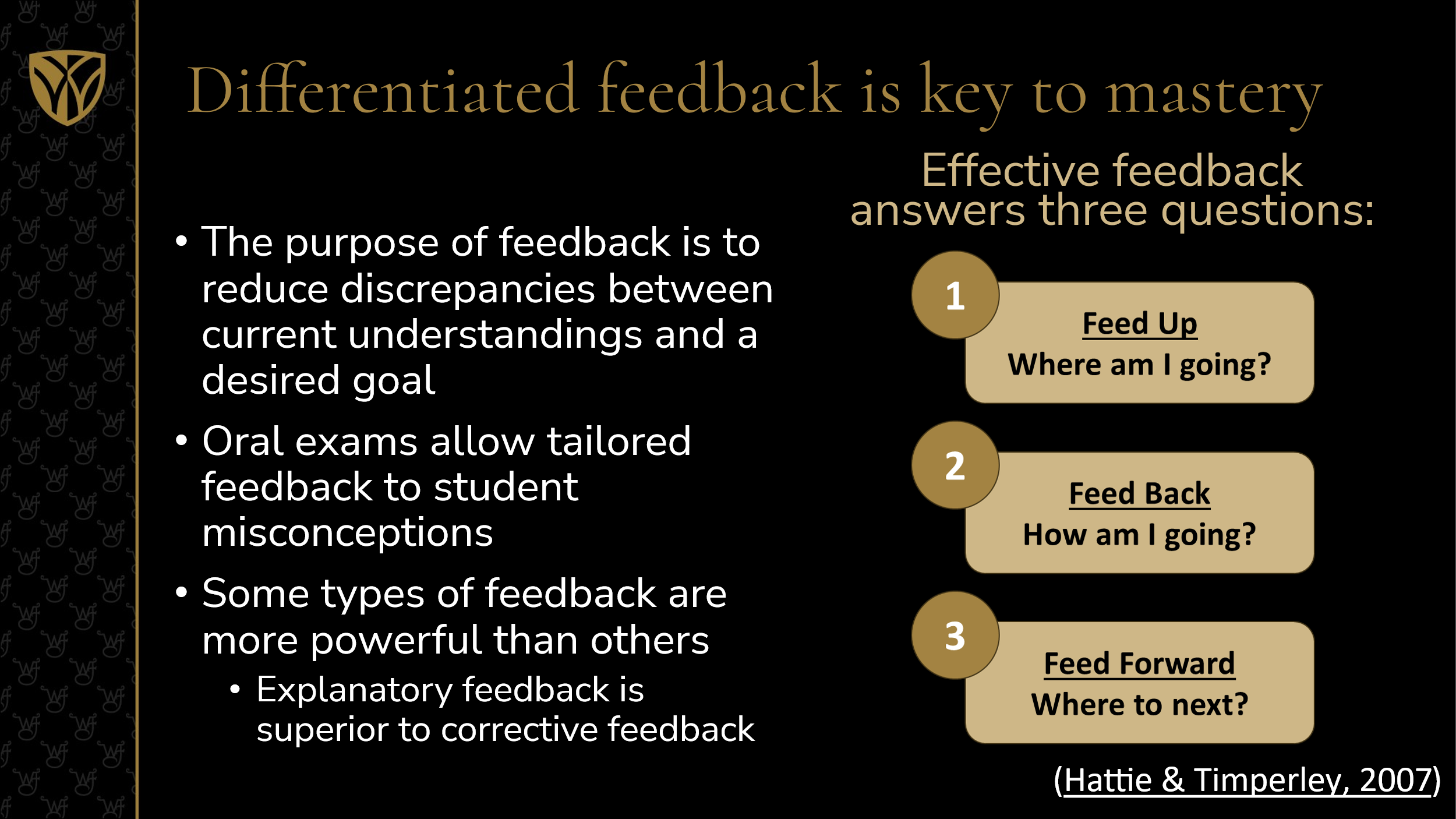


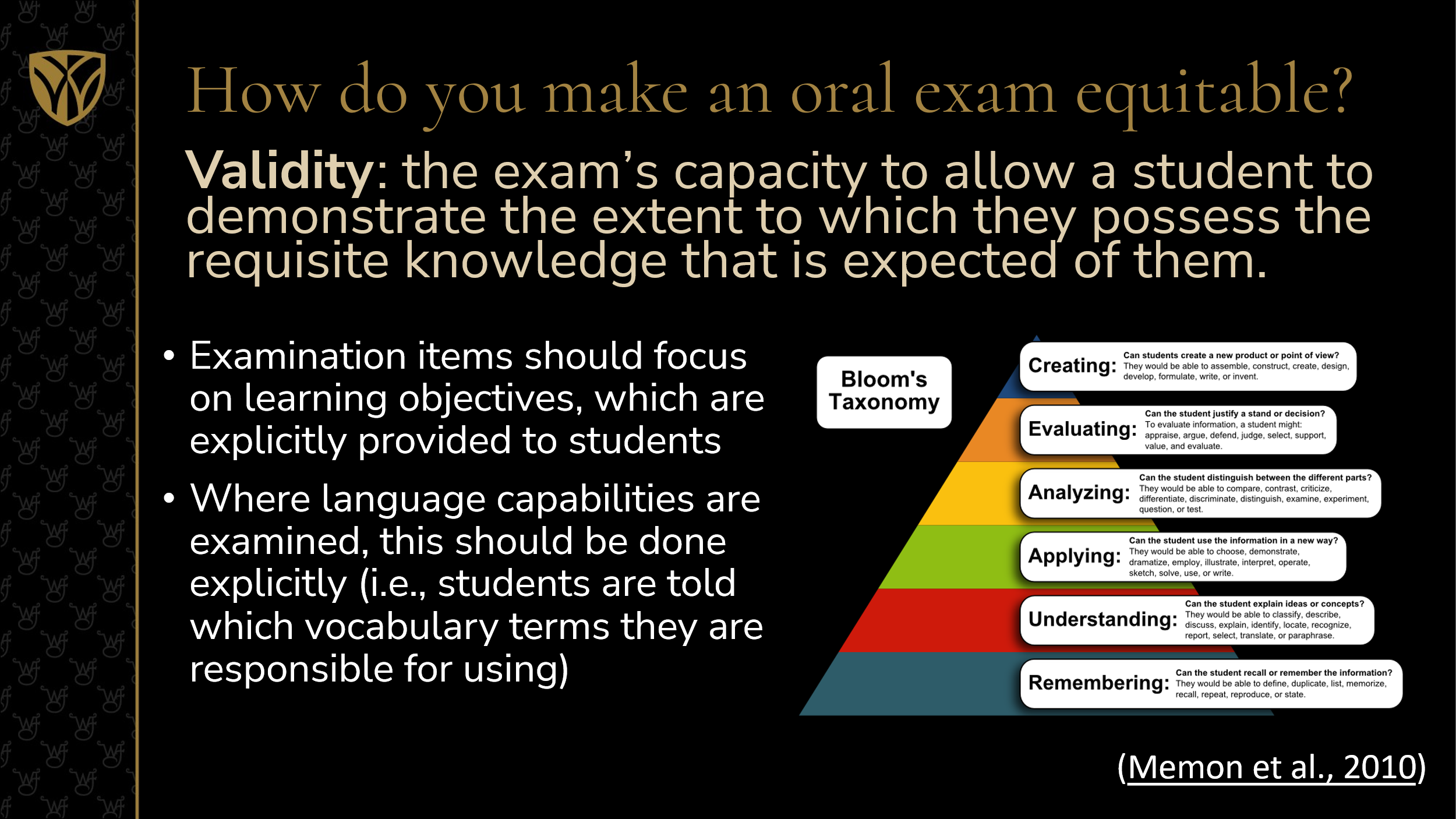


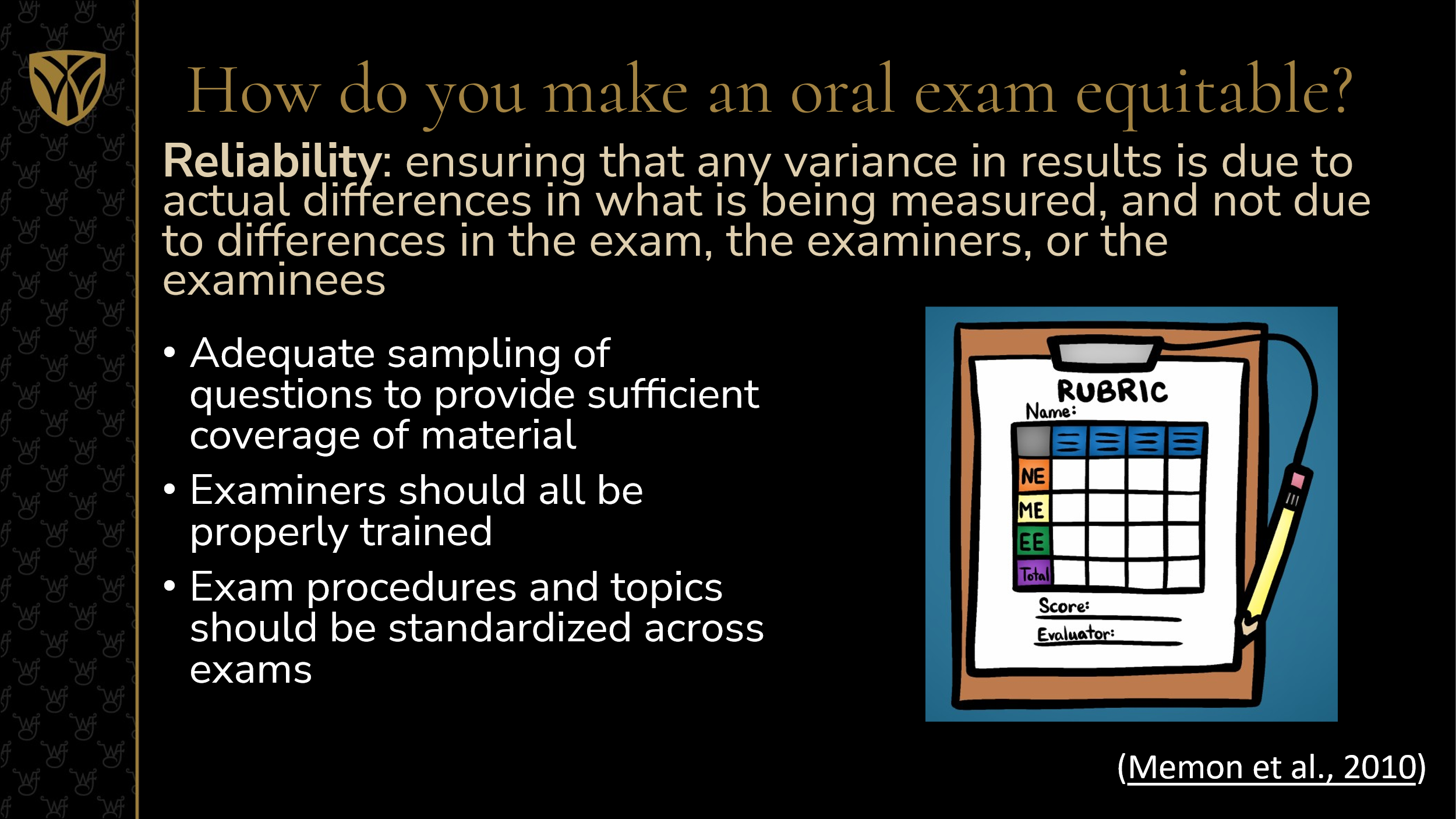


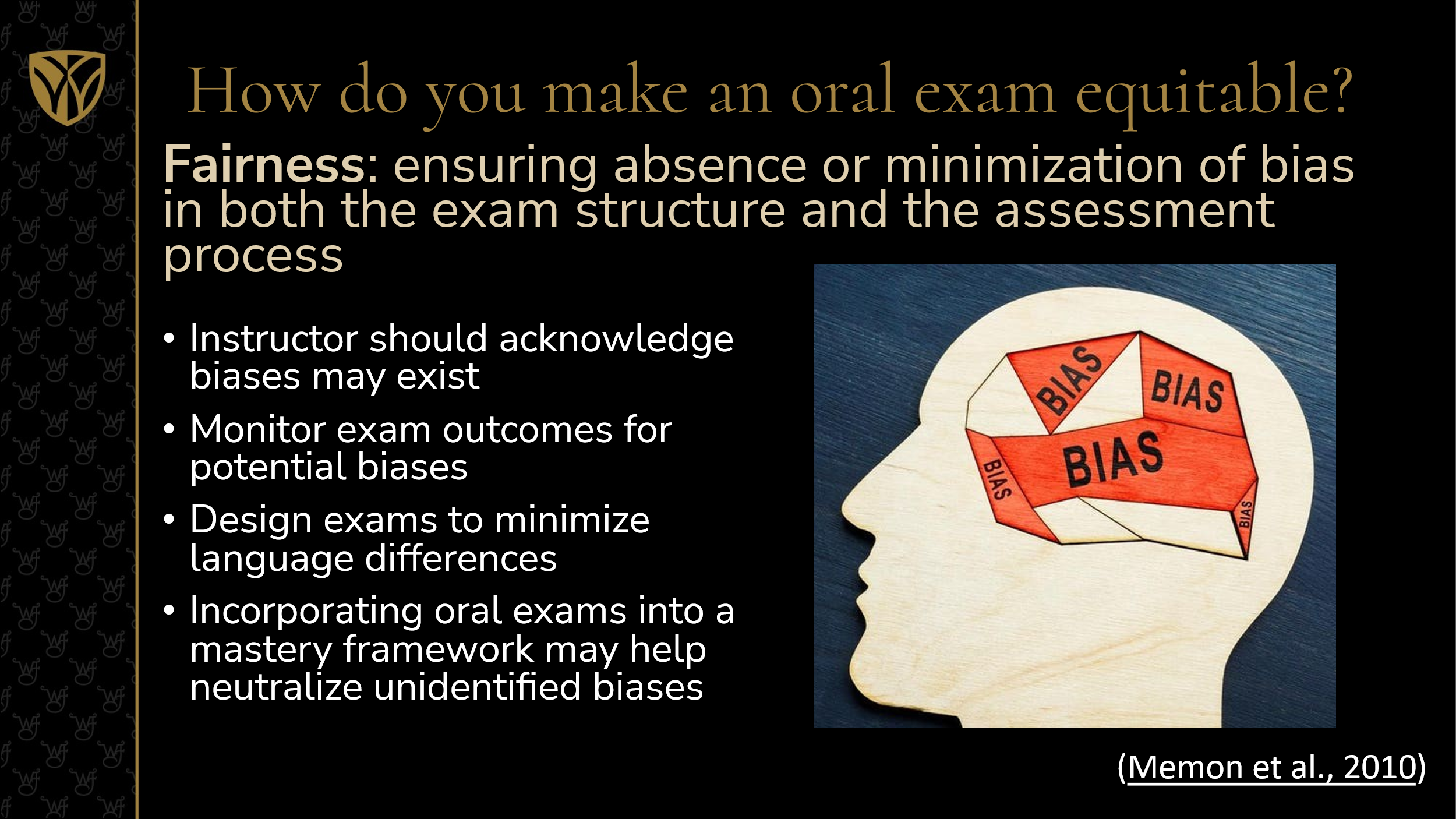


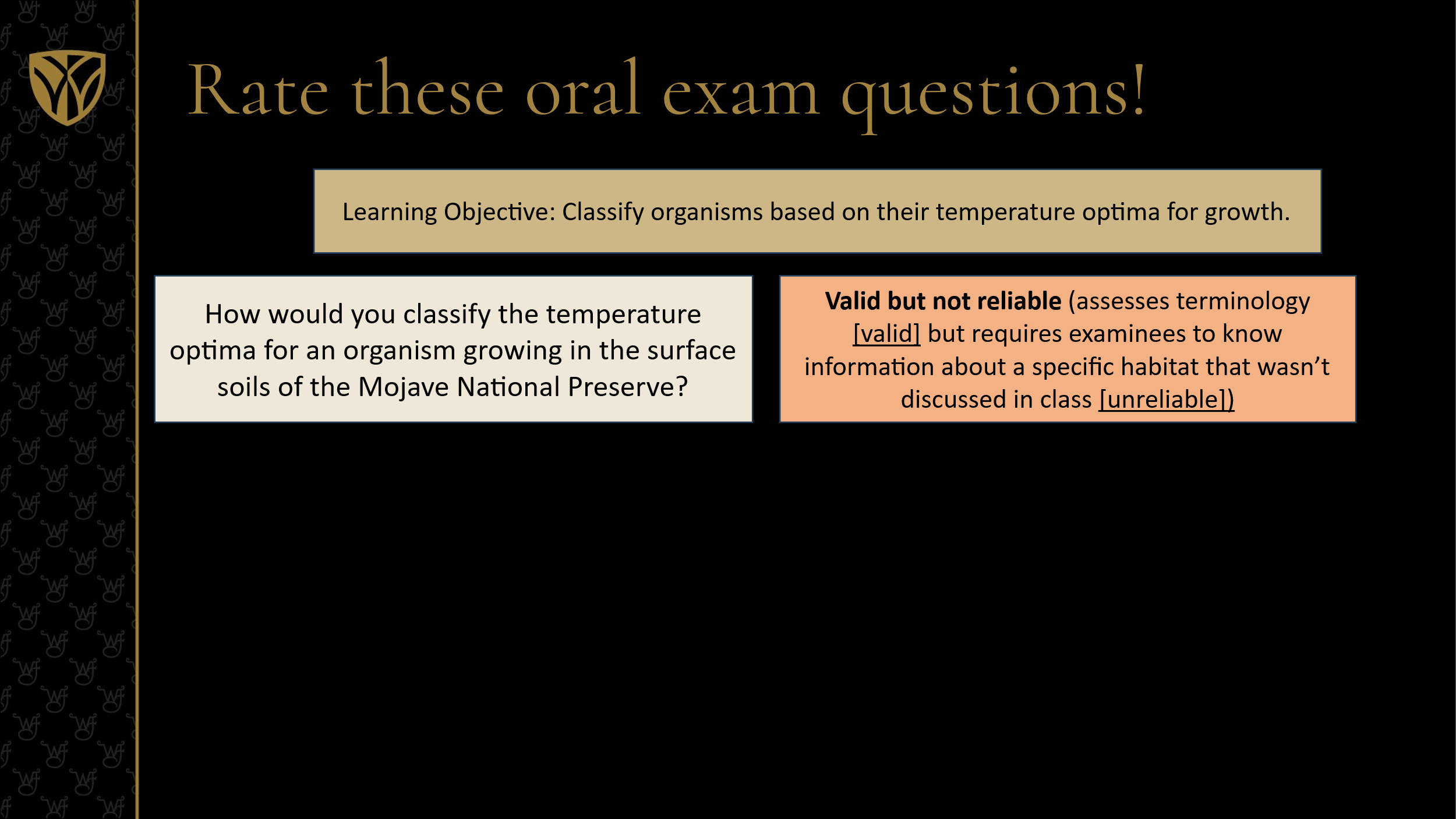


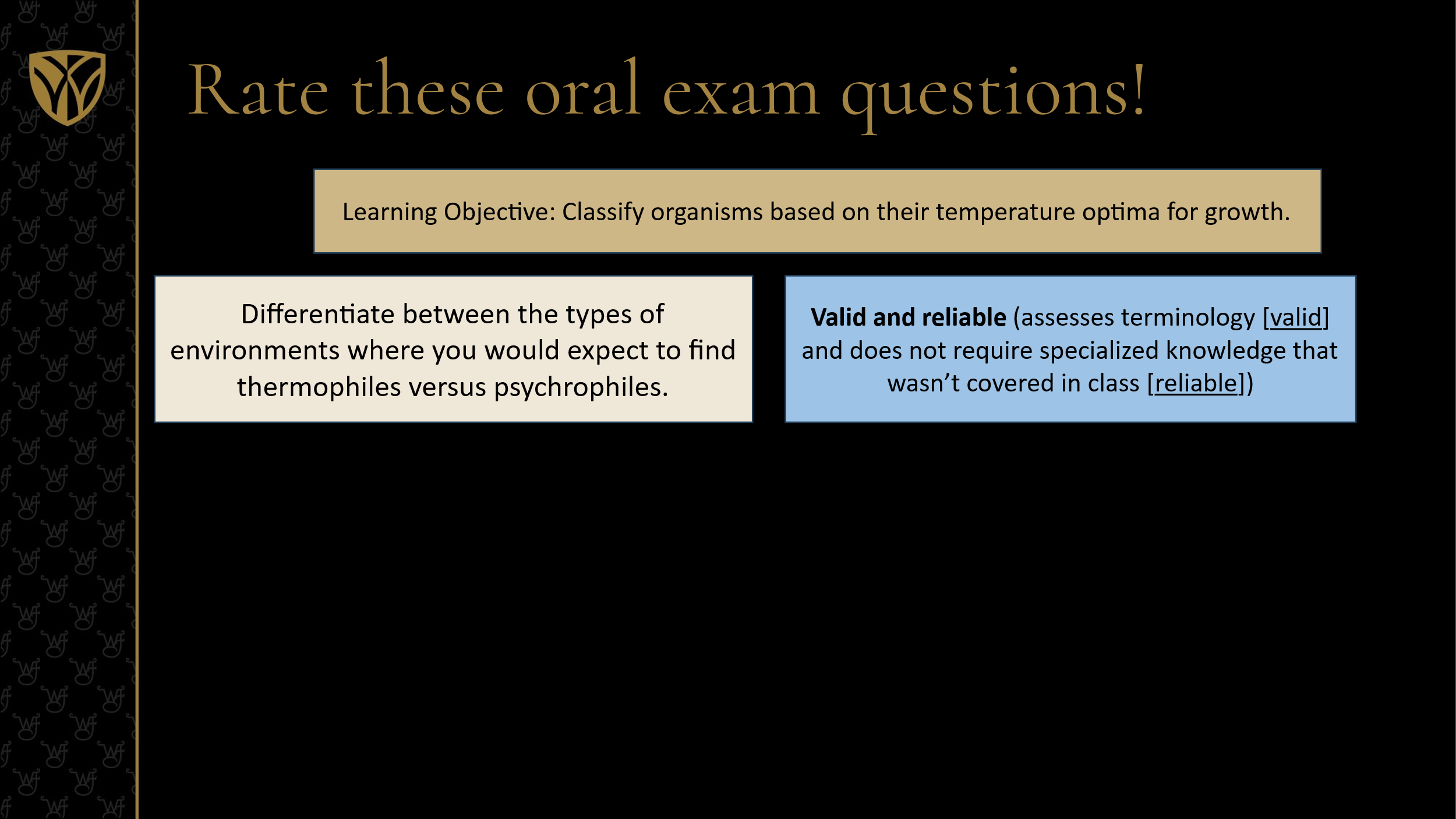


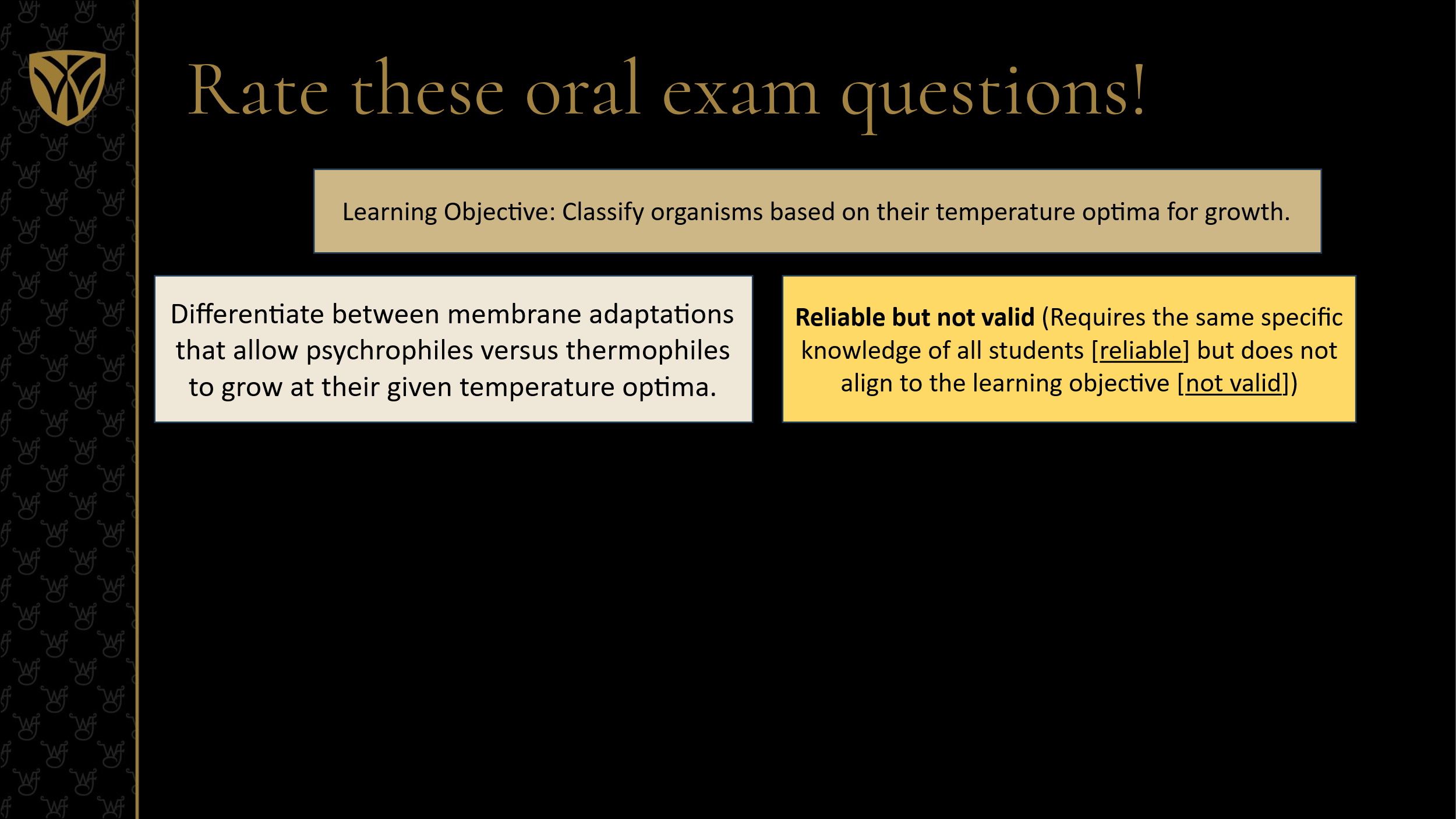


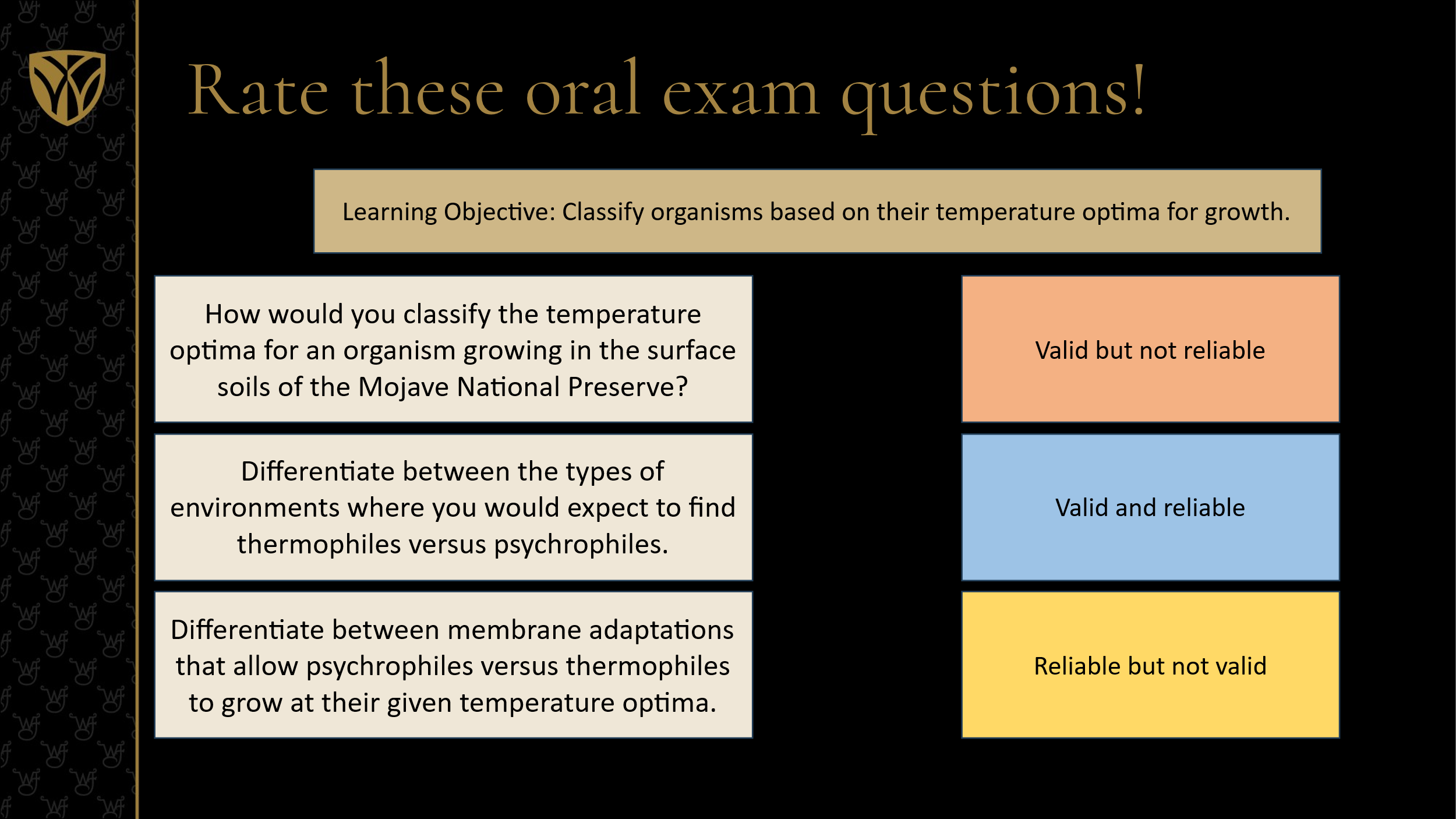


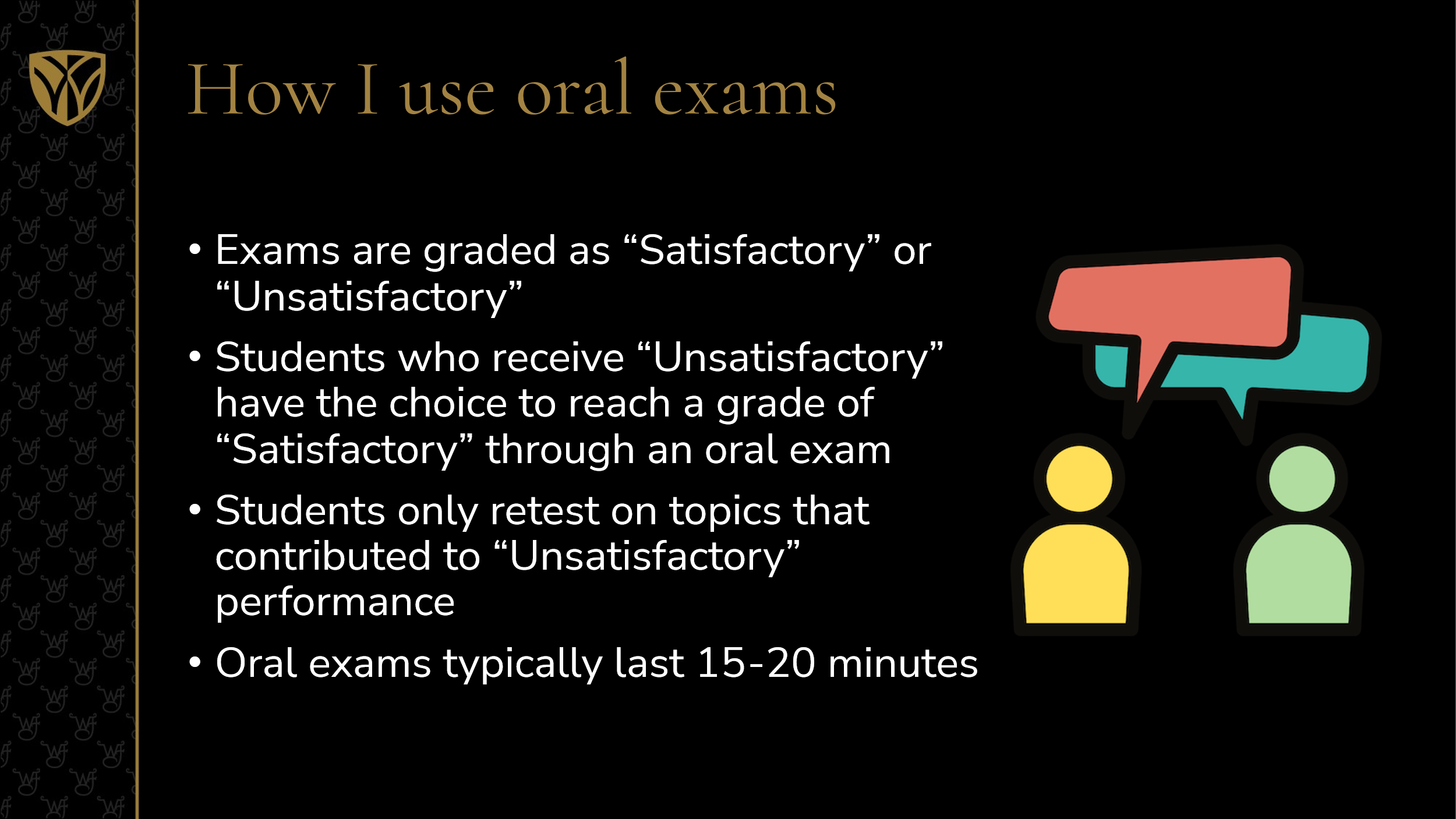


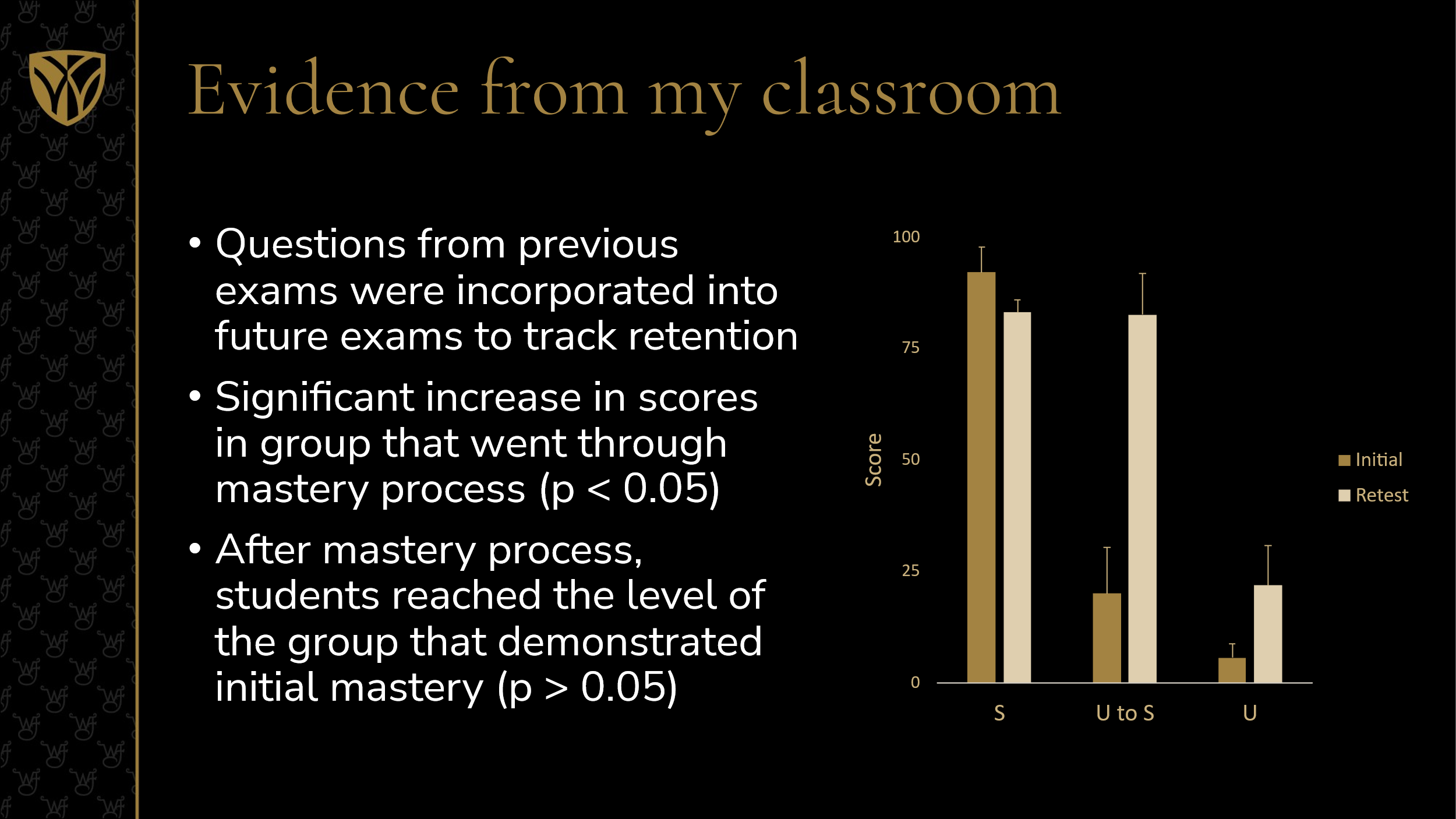


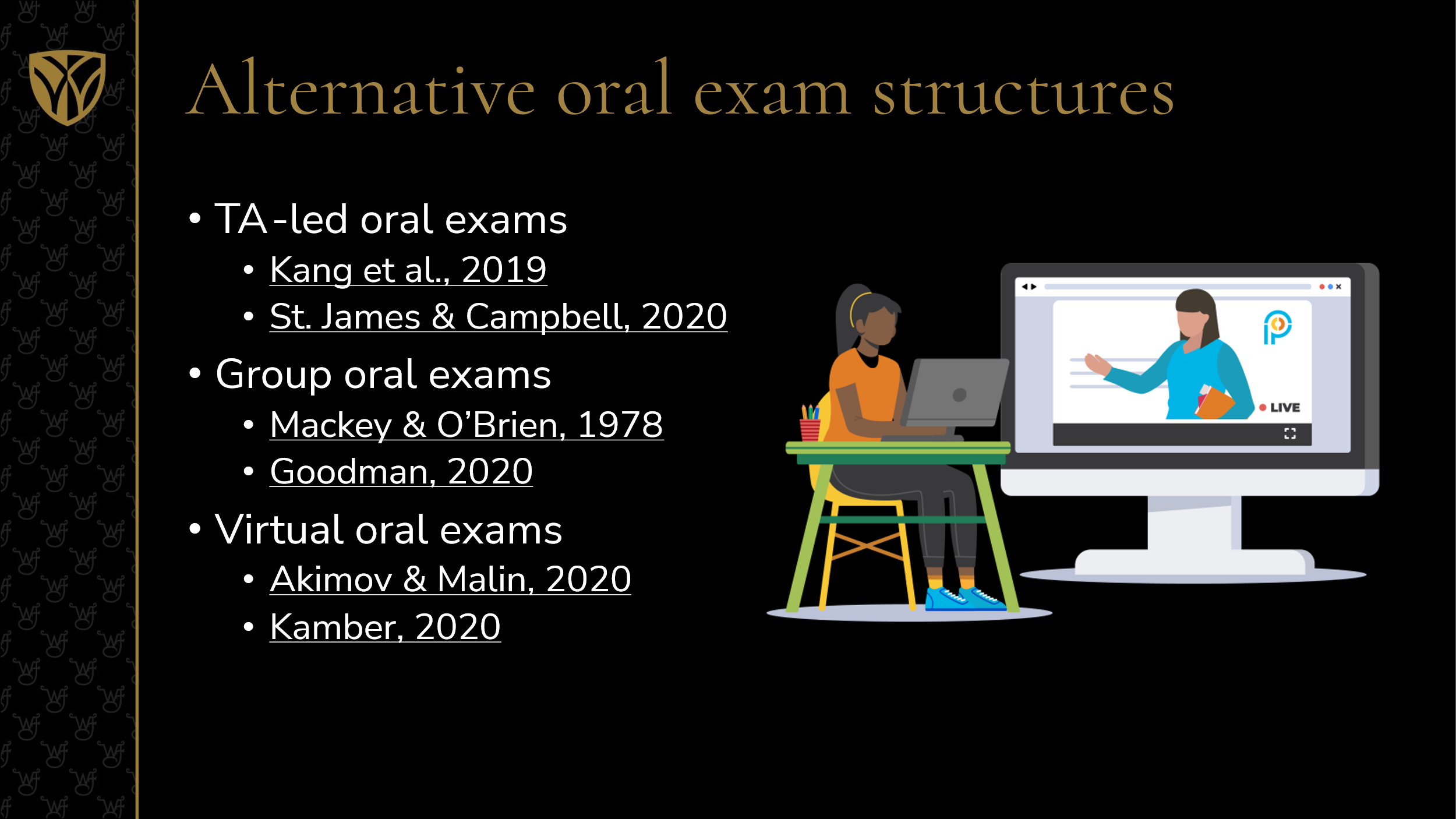


**Supplemental Text 2**: Sample scenarios from mock oral exams illustrating follow-up questions and feedback.

*Scenario #1: Illuminating Knowledge Transfer & Problem-Solving Approaches*

**Instructor:** In microbial systems, chemolithoautotrophy is common but chemolithoheterotrophy is extremely rare. Can you suggest an explanation why this “theoretically possible” class of physiology does not seem to be widespread?

**Student:** Because chemolithoheterotrophs get carbon from organic sources and energy from inorganic sources, while chemolithoautotrophs get carbon from CO_2_ and energy from inorganic sources.

**Instructor:** You’ve just highlighted some similarities and differences between chemolithoautotrophy and chemolithoheterotrophy. Can you state more explicitly what these physiologies share and how they differ?

**Student:** Both physiologies get their energy from inorganic molecules, but heterotrophs get their carbon from organic sources while autotrophs get their carbon from CO_2_.

**Instructor**: Can you suggest an explanation as to why this difference in carbon source could explain why chemolithoautotrophy is common but chemolithoheterotrophy is rare?

**Student:** Um… I’m not sure.

**Instructor:** If we were looking at chemoorganoheterotrophy, how would that organism get its carbon and energy?

**Student:** Both carbon and energy would come from organic compounds.

**Instructor:** The same organic compounds?

**Student:** Yes.

**Instructor:** So, organisms that take in organic compounds often have the ability to use them both as carbon sources and as energy sources?

**Student:** Yes.

**Instructor:** And, generally speaking, how would the amount of energy available in an organic compound compare with that in an inorganic compound?

**Student:** Organic compounds are better energy sources than inorganic compounds.

**Instructor:** With that in mind, why might chemolithoheterotrophy be rare?

**Student:** Oh, because if they were consuming organic carbon, it would make sense to also use it for energy generation because it could get more energy. So, it doesn’t really make sense to take up a good energy source and not actually use it for energy.

*Scenario #2: Providing Feedback*

**Instructor #1:** It looks like your biggest problem is connecting concepts of energy generation to classifying metabolic strategies. You should go back and review relative comparisons of energy yield from metabolic pathways so you’re better prepared when you return for a retest. (**BAD FEEDBACK**)

**Instructor #2:** The goal here is to be able to connect concepts of energy yield from different energy sources to the metabolic classification schemes microbiologists use. When you master this goal, you will understand why certain metabolic strategies are more widespread than others and why the balance of organisms with one physiology versus another varies from ecosystem to ecosystem. At the moment, you seem to have a decent understanding of the underlying concepts involved, but you need to work more on seeing the connections between these concepts. To be ready for a retest, I would advise that you build a concept map to try and illustrate the connections between energy yield and metabolic classification. Feel free to come to office hours after you’ve attempted this, and we can review your concept map and any other questions. (**GOOD FEEDBACK**)

**Supplemental Text 3**: Qualtrics Survey

Oral Exams Survey

Start of Block: Default Question Block

Q1 How did your anxiety around written tests in Microbiology compare to your anxiety in other classes?

- More anxiety about tests in Microbiology than in other classes (1)
- Similar anxiety about tests in Microbiology and in other classes (2)
- Less anxiety about tests in Microbiology than in other classes (3)

Display this question:

If How did your anxiety around written tests in Microbiology compare to your anxiety in other classes? = More anxiety about tests in Microbiology than in other classes

Q2 To what do you attribute your increased testing anxiety in Microbiology?

Display this question:

If How did your anxiety around written tests in Microbiology compare to your anxiety in other classes? = Less anxiety about tests in Microbiology than in other classes

Q3 To what do you attribute your decreased testing anxiety in Microbiology?

| Page Break |  |
| --- | --- |

Q4 Did you take any oral retakes throughout the semester?

- Yes (1)
- No (2)

Display this question:

If Did you take any oral retakes throughout the semester? = Yes

Display this question:

If Did you take any oral retakes throughout the semester? = Yes

Q6 What did you like or dislike about the oral exam retake process?

| Page Break |  |
| --- | --- |

Q7 I give consent for my answers and exam performance to be used to research purposes. (No identifying information will be reported and all answers and exam evaluations will remain confidential.)

- Yes (1)
- No (2)

End of Block: Default Question Block

**Supplemental Figures**


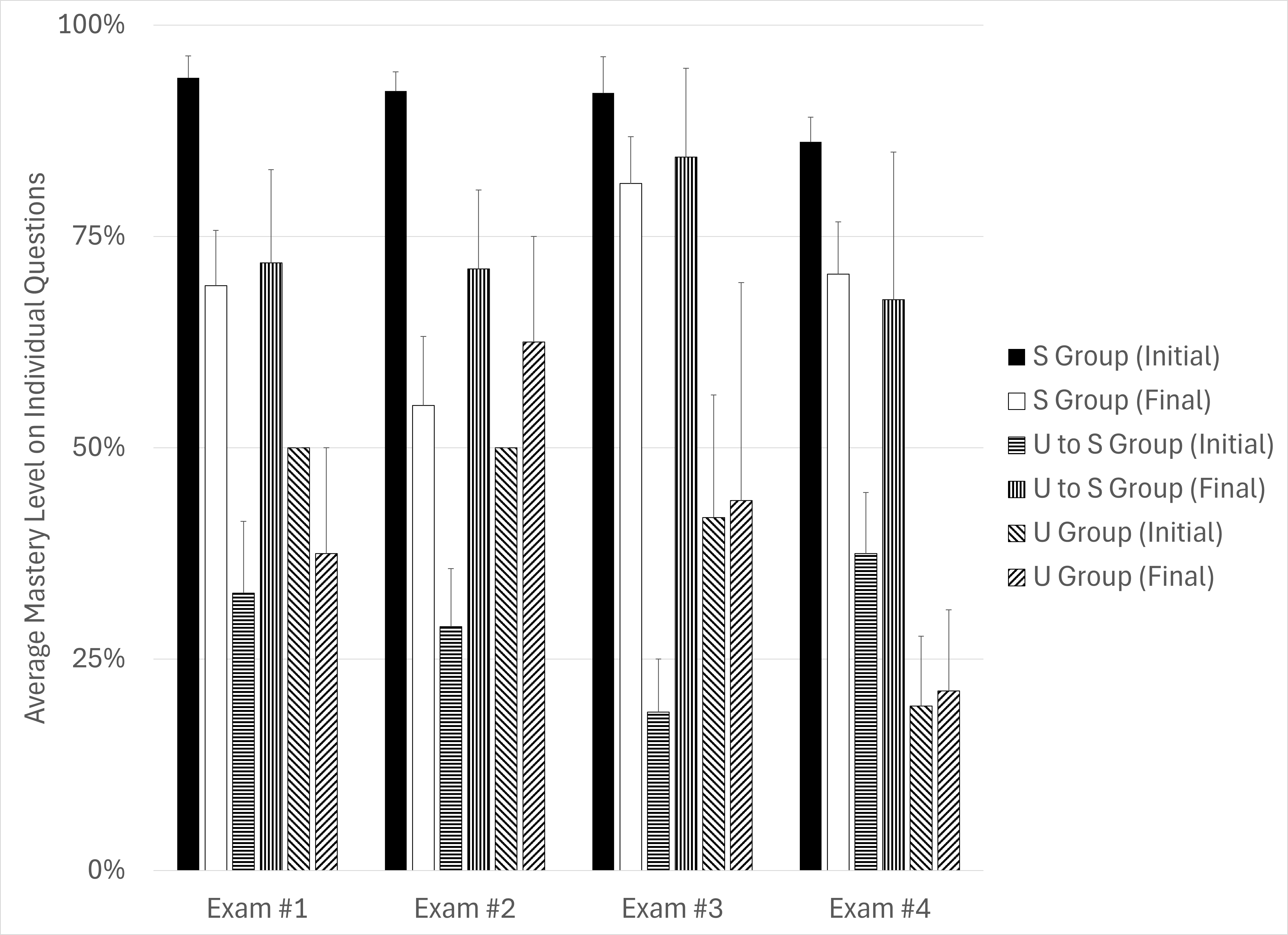


**Supplemental Figure 1**. Average mastery level on exam questions during initial assessment and final exam assessment for each module for students who initially mastered the material (S Group), initially performed poorly but mastered the material after the intervention (U to S group), and never mastered the material (U Group). Error bars represent standard error of the mean.


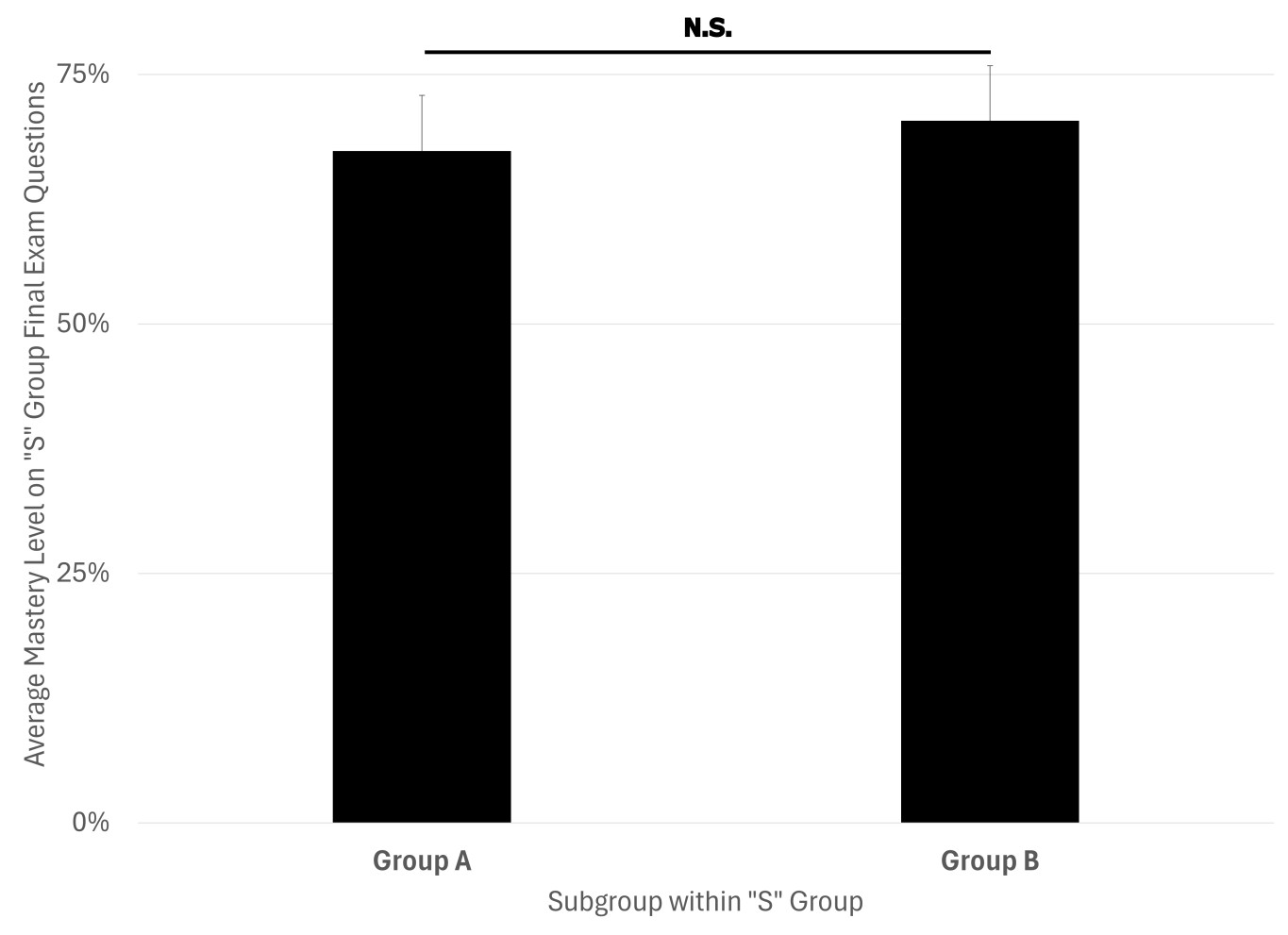


**Supplemental Figure 2**. Average mastery level during the final exam for students who initially mastered the material and did not participate in an intervention for the module (Group A) versus those who initially mastered the material and participated in an intervention for the module covering different but thematically related material (Group B). Error bars represent standard error of the mean. Significance levels: *p* < 0.05 (*),*p* < 0.01 (**), *p* < 0.001 (***), Not Significant (N.S.).


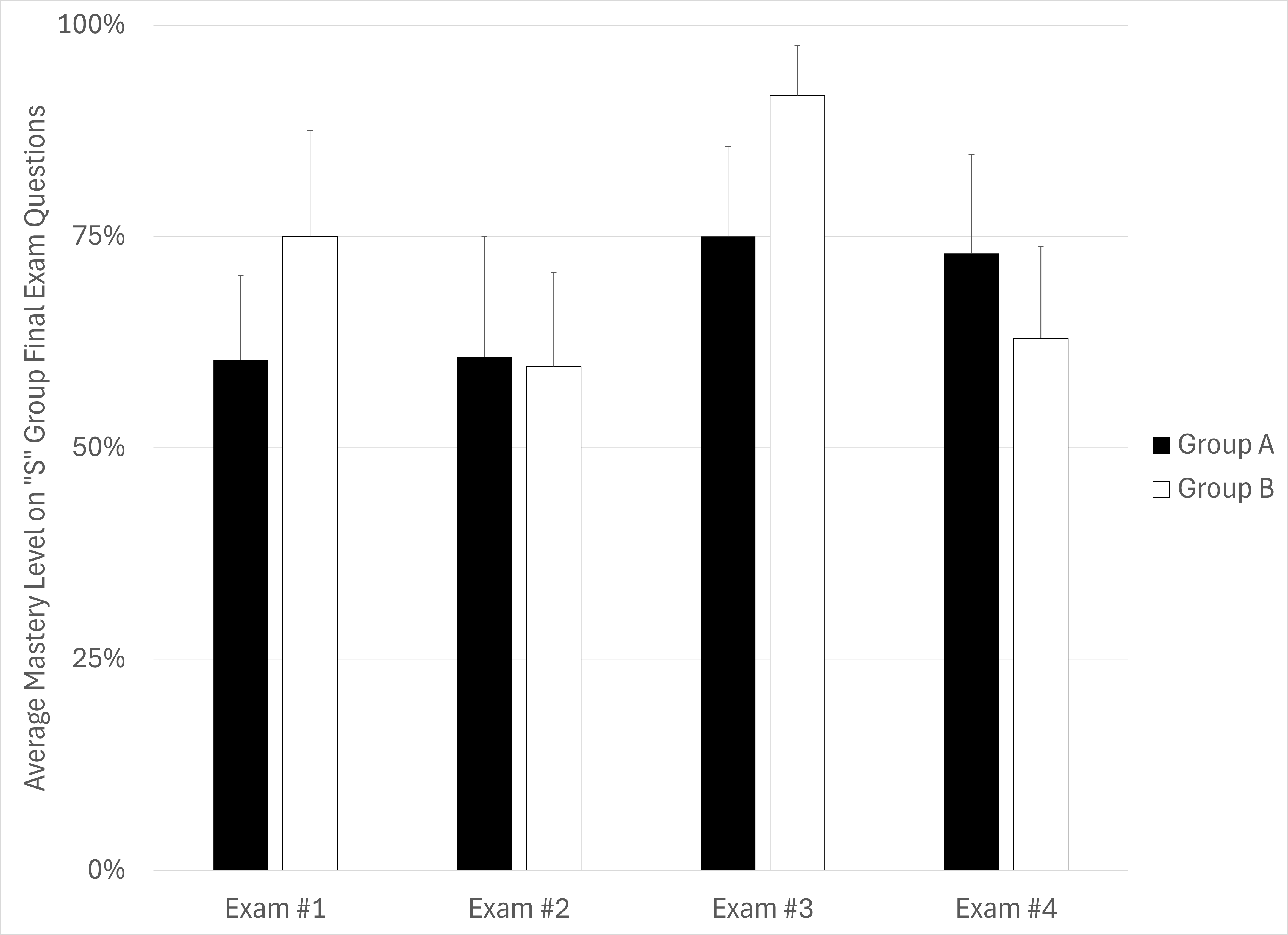


**Supplemental Figure 3**. Average master level during the final exam for each preceding module’s content for students who initially mastered the material and did not participate in an intervention for the module (Group A) versus those who initially mastered the material and participated in an intervention for the module covering different but thematically related material (Group B). Error bars represent standard error of the mean.

**Supplemental Tables**

**Supplemental Table 1**. Student responses to the question “To what do you attribute your increased testing anxiety in Microbiology?”

| **To what do you attribute your increased testing anxiety in Microbiology?** |
| --- |
| Studying and making extensive study guides to find difficult questions on the exams heightened my anxiety. Also, getting a chance at a U or F didn’t help as I felt more pressure on myself to get material right the first time. |
| General test anxiety, doing well in future plans (grad school etc), large amount of content to remember in detail |
| I’m not a great test taker and the Microbiology tests were much more detail specific than others |
| Difficult exams with a lot of preparation required |

**Supplemental Table 2.** Student responses to the question “To what do you attribute your decreased testing anxiety in Microbiology?”

| **To what do you attribute your decreased testing anxiety in Microbiology?** |
| --- |
| Knowing I could re-take the exams on areas I was weak on decreased my testing anxiety. I knew that my grade could be recovered because of the re-takes. Also knowing I just needed an S to do well significantly decreased my anxiety. |
| Having the the option to retake sections on the oral retake assessments for full credit |
| Retakes |
| The fact that I could explain verbally instead of writing and I had a chance to focus in on what I did not know |
| Dr. St James’ openness to answering questions, helping and accommodating students. Our ability to choose when to take our tests really helped me in times when I had busy weeks and my studying would not have been as beneficial. |
| Being able to retake if we messed up some concepts. Also being able to retake quickly through Interview style questions |
| The ability to do oral retakes and the module exams instead of big cumulative exams |
| The guided notes and study guide you release for each module |
| Proper studying and organization of content made it easy to feel prepared. The retake also provided a safety net. |

**Supplemental Table 3**. Student responses to the question “What did you like or dislike about the oral exam retake process?”

| **What did you like or dislike about the oral exam retake process?** |
| --- |
| They were lowkey and less stressful. |
| It was nerve racking to have to do an oral retake, as the stakes felt higher than a written exam. But from my one oral retake, I found that I slightly liked them better as I could early explain the concepts I missed. |
| Sometimes it was still anxiety inducing but overall helpful. |
| That if I was not understanding a question, he reworded it so that I could better grasp what exactly he wanted me to answer |
| I am more of a writer exam taker but apprwciated the ability to retake and explain |
| I loved it. Needs to be applicable for every exam. I did loose some internal motivation in studying for the first time though knowing that I had a retake waiting for me |
| Liked it all, thought it was fair and help me accountable |
| I liked that they were interview style questions that gave me an ability to show what I know |
| I was not entirely sure how in depth to go about my knowledge of the content at first |
| I think my knowledge finally was really expressed when I started doing them |
| I liked that it allowed me to fully explain what I knew about the topic. Lots of test questions I got wrong was because I didn't understand the question, but I was able to demonstrate that I knew the material through oral retakes |
| I disliked this process because it was very stressful but I think that it was a good way to demonstrate my effort and knowledge of the course material |
| It was much quicker and informal than I feared it would be |
| N/A |
| I thought they were very helpful and actually made me learn and understand the concepts. |
| I thought they were quick and to the point which I appreciated |
| I liked how I could show what I knew in words instead of having to write it on paper |
| It was really intimidating one on one |
